# Using Machine Learning to Predict Synergistic Antimalarial Compound Combinations With Novel Structures

**DOI:** 10.3389/fphar.2018.01096

**Published:** 2018-10-02

**Authors:** Daniel J. Mason, Richard T. Eastman, Richard P. I. Lewis, Ian P. Stott, Rajarshi Guha, Andreas Bender

**Affiliations:** ^1^Department of Chemistry, Centre for Molecular Informatics, University of Cambridge, Cambridge, United Kingdom; ^2^Healx Ltd., Cambridge, United Kingdom; ^3^Division of Preclinical Innovation, National Center for Advancing Translational Sciences, National Institutes of Health, Rockville, MD, United States; ^4^Unilever Research and Development, Wirral, United Kingdom

**Keywords:** synergy, combinations, malaria, plasmodium falciparum, artificial intelligence, modeling

## Abstract

The parasite *Plasmodium falciparum* is the most lethal species of Plasmodium to cause serious malaria infection in humans, and with resistance developing rapidly novel treatment modalities are currently being sought, one of which being combinations of existing compounds. The discovery of combinations of antimalarial drugs that act synergistically with one another is hence of great importance; however an exhaustive experimental screen of large drug space in a pairwise manner is not an option. In this study we apply our machine learning approach, Combination Synergy Estimation (CoSynE), which can predict novel synergistic drug interactions using only prior experimental combination screening data and knowledge of compound molecular structures, to a dataset of 1,540 antimalarial drug combinations in which 22.2% were synergistic. Cross validation of our model showed that synergistic CoSynE predictions are enriched 2.74 × compared to random selection when both compounds in a predicted combination are known from other combinations among the training data, 2.36 × when only one compound is known from the training data, and 1.5 × for entirely novel combinations. We prospectively validated our model by making predictions for 185 combinations of 23 entirely novel compounds. CoSynE predicted 20 combinations to be synergistic, which was experimentally validated for nine of them (45%), corresponding to an enrichment of 1.70 × compared to random selection from this prospective data set. Such enrichment corresponds to a 41% reduction in experimental effort. Interestingly, we found that pairwise screening of the compounds CoSynE individually predicted to be synergistic would result in an enrichment of 1.36 × compared to random selection, indicating that synergy among compound combinations is not a random event. The nine novel and correctly predicted synergistic compound combinations mainly (where sufficient bioactivity information is available) consist of efflux or transporter inhibitors (such as hydroxyzine), combined with compounds exhibiting antimalarial activity alone (such as sorafenib, apicidin, or dihydroergotamine). However, not all compound synergies could be rationalized easily in this way. Overall, this study highlights the potential for predictive modeling to expedite the discovery of novel drug combinations in fight against antimalarial resistance, while the underlying approach is also generally applicable.

## Introduction

Malaria is a deadly and worldwide disease, with an estimated 445,000 deaths globally in 2016, of which 91% are estimated to have occurred in Africa (World Health Organisation, 2017). Despite global mortality rates declining by 62% between 2000 and 2015, this disease remains a major killer for children under 5 years, with a young life being taken every 2 min (World Health Organisation, 2017).

**Figure 1:**
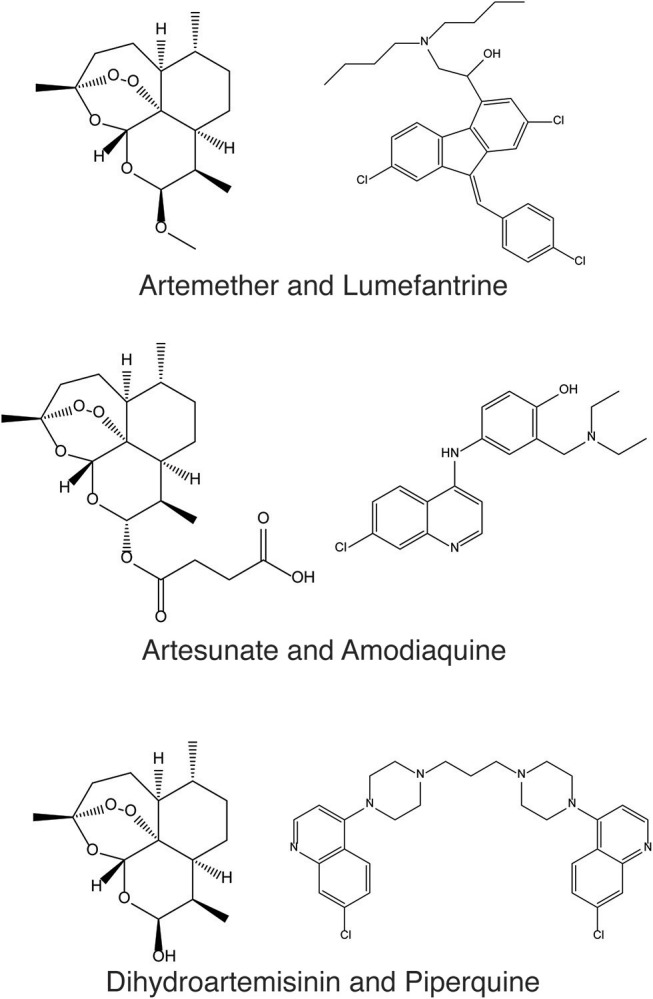
Artemether and Lumefantrine, Artesunate and Amodiaquine, and Dihydroartemisinin and Piperaquine are antimalarial combinations recommended by the WHO as the current standard of care to help protect against drug resistance in *P. falciparum*.

When exposed to antimalarial compounds, the malaria-causing parasite *Plasmodium falciparum* can over time develop resistance to different therapies and *via* a number of distinct mechanisms (Mita and Tanabe, 2012). This tendency has rendered many antimalarial therapies ineffective in the past, and continues to threaten the current standards of care. In order to combat resistance, options include the design or discovery of new antimalarial compound classes or analogs that offer increased efficacy over those with prior use. However, in the present time, and in absence of these novel discoveries, the current World Health Organization (WHO) guidelines state that combinations of at least two effective antimalarial medicines with different modes of action need to be administered in order to help protect against resistance (World Health Organisation, 2015). At present, the standard of care listed by WHO includes artemisinin-based combination therapies (ACT), such as artemether with lumefantrine, artesunate with amodiaquine, and dihydroartemisinin with piperaquine (Figure 1). Resistance to artemisinins has arisen more recently in South East Asia (World Health Organisation, 2017), raising concern on the future effectiveness of ACTs since resistance to the ACT partner drug significantly decreases the clinical efficacy of the combination therapy (Bacon et al., 2007). Alarmingly, this concern has recently been confirmed in Cambodia, in the form of resistance to the first line treatment dihydroartemisinin-piperaquine by *P. falciparum* strain *PfPailin* (Imwong et al., 2017). The evolution and spread of multidrug resistant organisms renders the selection of novel drug combinations only a viable medium-term option, and there is continued effort to map ACT partner drugs by the World Wide Antimalarial Resistance Network (World Wide Antimalarial Resistance Network, 2014).

The combined properties resulting from a mixture of drugs is not always equivalent to the sum of their parts. Drug combinations are well-known to result in an increase or decrease in measured therapeutic efficacy (synergy or antagonism, respectively), result in no difference in effectiveness (additivity), or present an increase or decrease in the number of side effects experienced (drug-drug interactions, which would then also possibly represent synergy, albeit of undesired effects; Lehár et al., 2009; Tatonetti et al., 2012). In the case of malaria (and probably many other diseases one wants to treat), the desired effect sought after is usually synergy, i.e., a drug combination for which the antimalarial effect is greater than that observed by each compound alone, and greater than what would be expected by assuming solely additivity of compound effect (Sucher, 2014). In this case lower doses of each individual compound would be required, thereby potentially achieving the desired efficacy with in many cases reduced side-effects (Csermely et al., 2005).

Antimalarial drug combinations can be either novel, or represent the repurposing of drugs used previously for other purposes, such as in the use of tricyclic antidepressants in chloroquine-resistant strains of *P. falciparum* (Bitonti et al., 1988). High throughput screening for antimalarial compound combinations is one mechanism by which discovery of novel combinations may be found faster (Mott et al., 2015). However, the discovery of synergistic combinations is experimentally challenging: As the number of compounds increases, very quickly too does the number of potential combinations, in particular when considering multiple replicates, the requirement of screening concentration matrices, and possibly against different strains of the pathogen. For example, 100 compounds screened pairwise results in 4,950 compound combinations, and testing for synergy in a 6 × 6 dose-response matrix altogether requires 178,200 data points (with numbers increasing further when taking into account replicates, different strains, etc.; Cokol et al., 2014). Increasing the search space by the addition of just 25 more compounds would require over 100,000 further data points, due to combinatorial explosion.

Computational approaches have been investigated as a means to predict the synergistic interaction of compounds previously, with methods that utilize networks of pathways and simulation (Lehár et al., 2007; Nelander et al., 2008; Miller et al., 2013; Huang et al., 2014; Patel et al., 2014; Zhang et al., 2014), relationships between physicochemical properties (Yilancioglu et al., 2014), chemogenomics approaches (Bansal et al., 2014; Wildenhain et al., 2015; KalantarMotamedi et al., 2018), and single agent efficacies (Gayvert et al., 2017) and/or combinations (Menden et al., 2018) measured across multiple cell lines (for recent reviews of compound combination modeling and perspectives, see Bulusu et al., 2016; Weinstein et al., 2017; Tsigelny, 2018). A disadvantage to many of these approaches is that they often require experimental knowledge of underlying biological interactions between drugs and disease, or chemogenomic or phenotypic readouts (Jansen et al., 2009; Bansal et al., 2014; Wildenhain et al., 2015; Menden et al., 2018). This data may be difficult to obtain, non-existent, or expensive to collect enough to create a predictive model from. In addition, the prediction of novel combinations themselves will rely on the same experimental descriptors being available for each new compound.

**Figure 2:**
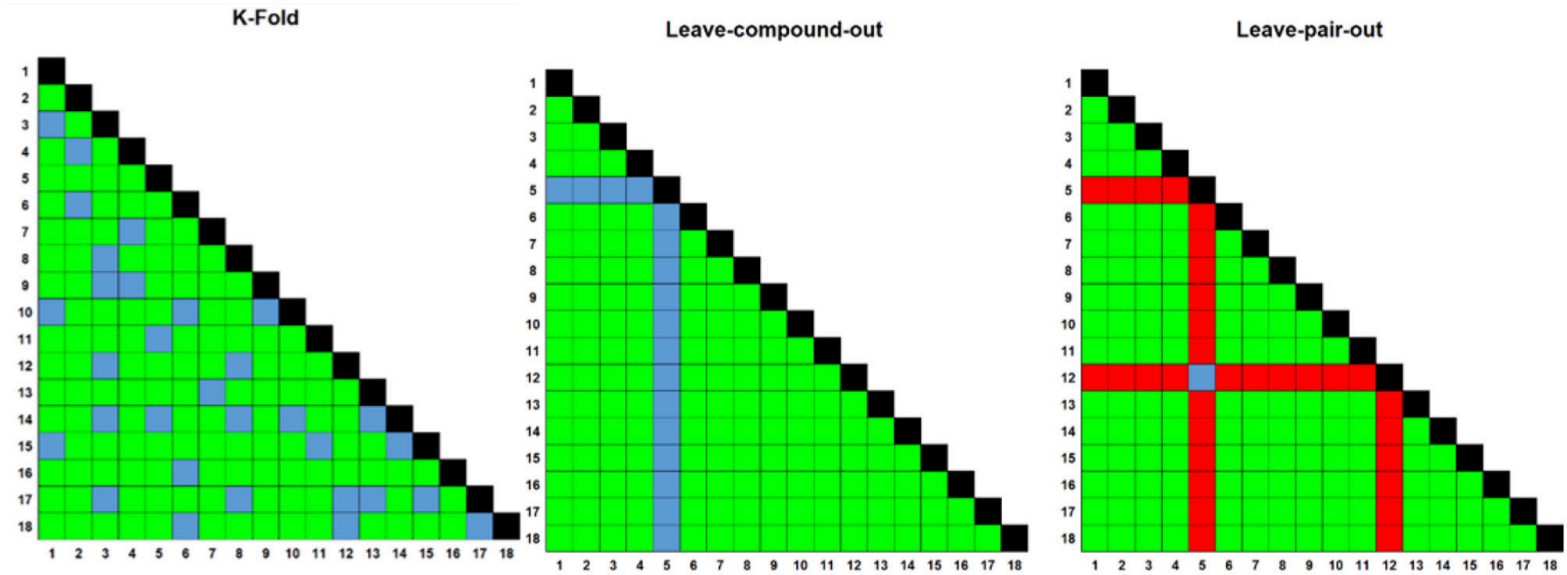
Three different rounds of cross-validation (CV) were employed to test model performance prior to making final predictions. Numbers on axes represent compound IDs in a compound combination training dataset. K-fold randomly selects a 1/K fraction of combinations to remove from the training data and predict in each round; Leave One Compound Out (LOCO) chooses pairs to remove based upon one compound in each round, and Leave One Pair Out chooses pairs to remove based upon a choice of two compounds in each round. Green; training combinations; blue; test combinations, red; held-out combinations, black; self-self crosses (not included in training data).

In order to address these problems, we have developed CoSynE (Combination Synergy Estimation; Mason et al., 2017). CoSynE constructs predictive models from existing combination screening data, and utilizes only the known structures of compounds that have been part of these screens. As such, CoSynE requires only two pieces of information, namely a list of compounds together with their structural representations, and a list of compound combinations together with a label whether the action of each combination was found to be synergistic, antagonistic, or additive (depending on the criteria for those categories one finds appropriate in a particular case). The compounds are transformed into two classes of representation by CoSynE: Firstly, a compound structure fingerprint (SFP; a 2048-bit Morgan Fingerprint), and secondly a predicted target fingerprint representing bioactivity spectra [TFP; 1,080 predicted protein target binding probabilities above a training cut-off, using PIDGIN (Mervin et al., 2015)]. This hence yields three classes of models: SFP, TFP, and STFP (a concatenation of the SFP and TFP fingerprints). These fingerprints are used as input to machine learning models that make inferences between a particular representation and the experimentally observed synergy. A number of models are optimized for the prediction of synergistic combinations, and the best-performing final model is selected following a rigorous cross-validation procedure, where either both compounds are known to the model, one compound is unknown, or both are unknown, such that the ability of CoSynE to extrapolate to novel chemical space may be inferred (Figure 2).

We have previously applied CoSynE to the prediction of novel antibiotic combinations effective against *E. coli* (Mason et al., 2017). In this initial study, CoSynE was trained upon 156 pairs of 18 compounds using the SFP representation of combinations (since in preliminary studies other types of descriptors were found to lead to inferior performance), which was then used to pre-screen a set of 123 combinations, comprising compounds that were known and/or unknown to the model. After prospective validation, 10 novel synergistic combinations were confirmed from a list of 12 that were highlighted by CoSynE. The results from our previous study correspond to a 2.8-fold enrichment in the discovery of synergistic combinations vs. that expected by random selection from the same set of compounds.

In the present study, we were starting with a much larger training dataset consisting of 1,540 combinations of 56 compounds tested against *P. falciparum* (Mott et al., 2015). Next, CoSynE was used to pre-screen a library of 23 compounds *unknown to the model* (see Methods section for compound selection process) by predicting which combinations of those compounds are likely to exhibit novel antimalarial synergy.

These predictions were prospectively validated by carrying out a full pairwise experimental screen of all 23 compounds the model could have chosen from (in order to also provide a negative control, i.e., testing of compound combinations not predicted to be synergistic by the model). This validation represents making predictions in entirely novel compound space, where both compounds have not been seen by the model before, which is a very tough challenge, compared to our previous study (and many other studies) which mostly included compounds that were previously known to the model. However, prospective validation in the present study showed CoSynE predictions to be enriched with 1.70 times more synergistic combinations than expected by random selection (over an already rather high baseline synergy level, see details below), and hence also predictions in novel chemical space are enriched over random.

## Results and discussion

### Similarity of training and validation sets

**Figure 3:**
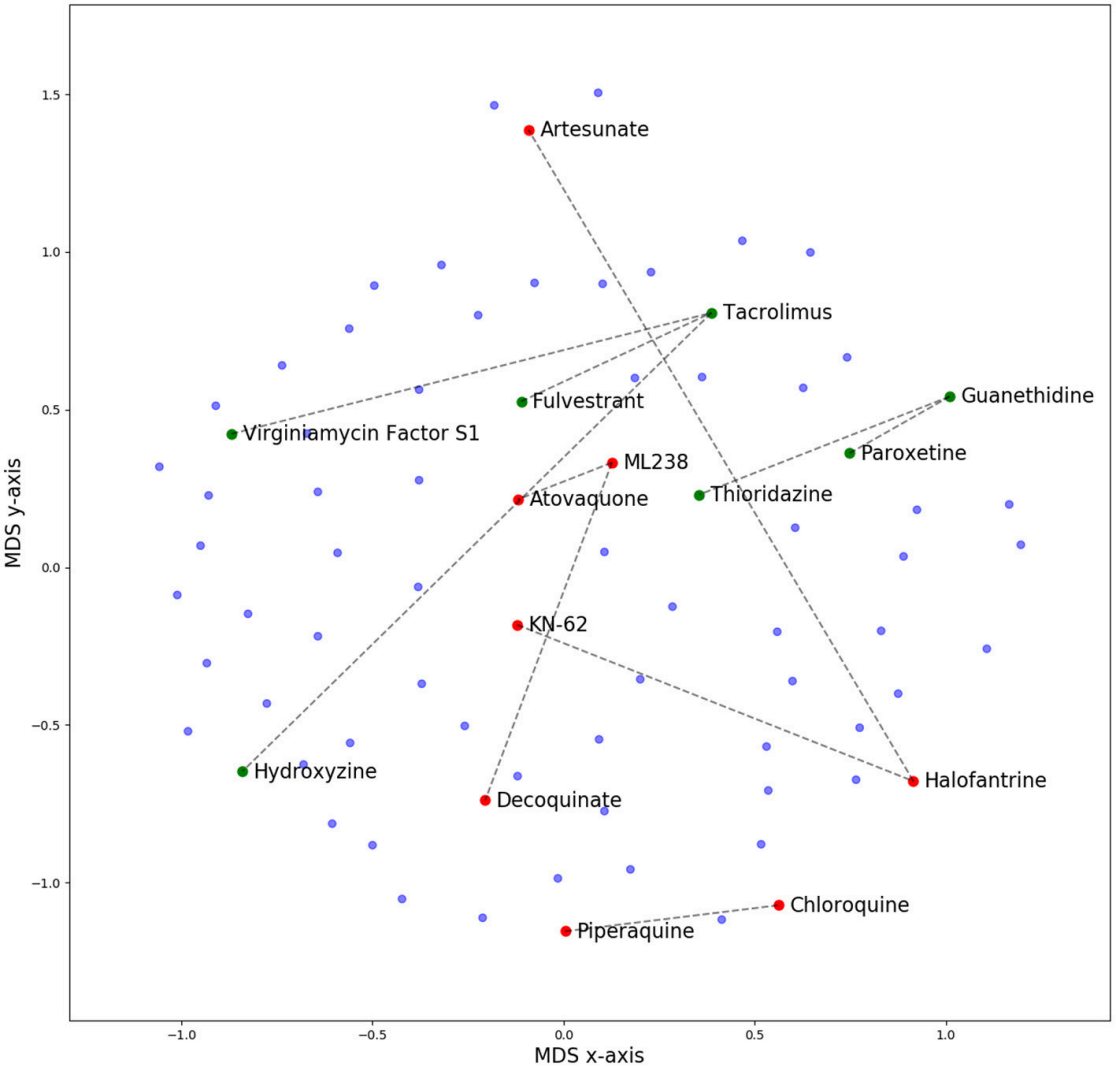
Multi-Dimensional Scaling (MDS) plot of chemical space for all compounds used in this study, based upon pairwise similarity of radius 2, 2,048-bit Morgan fingerprints. Compounds that comprise the top five synergistic combinations in the training (red dots) and prospective validation (green dots) datasets are highlighted, together with their synergistic connection. The lack of a clear clustering suggests that pairs of synergistic compounds do not always arise from those in distinct or well-defined chemical space. Out of these predictions in green, none were predicted by CoSynE, but paroxetine + guanethidine would be discovered following the indirect route described in the Results section, and is the second-most synergistic combination in the validation dataset. Structures for validation and training compounds are included in Supplementary Tables 5, 7, respectively.

Clustered hierarchical similarities are shown for whole and scaffold structures in Supplementary Figure 1. In general, there is little structural similarity between compounds in the training data compared to the prospectively tested data. Compounds which formed the top five most synergistic combinations in both the training and validation datasets are shown in dimensionally-reduced chemical space in Figure 3. The lack of a clear clustering between the top synergistic compound structures in either datasets demonstrates the difficulty in selection of compounds to screen simply *via* structural similarity alone. In addition to the observation that synergy is more commonly observed for drugs targeting the same processes (Brochado et al., 2018), the relationship between compound structure-related properties and synergistic interaction has been shown previously [such as lipophilicity and synergy in the case of anti-fungals (Yilancioglu et al., 2014)]. Overall, the inference of complex relationships, such as these on a scale that may quickly explode to intractable proportions is a task highly applicable to machine learning.

### Dataset composition and model performance during cross-validation on training set

The number of high quality (HQ) training combinations per dataset (see Methods section for definition) and synergy type is shown in Table 1. The Dd2 dataset contains the greatest number of HQ combinations (1,245), followed by 3d7 (1,194), and then Hb3 (1,159). This was reflected in the results of the 5-fold leave-one-compound-out (LOCO) and leave-one-pair-out (LOPO) cross-validation routines (Supplementary Table 1), which showed the Dd2 model to outperform 3d7 and Hb3. The mean average Matthews Correlation Coefficient (MCC) score for each strain (i.e., across all fingerprint type and all CV routines) were 0.19 (Dd2), 0.18 (3d7), and 0.11 (Hb3). Although these MCC scores are not particularly high in absolute terms (particularly since the more difficult CV routines bring the scores down, while considering that a score of 0 is equivalent to random selection), the Dd2 dataset was chosen for use in the remainder of the study due to the expectation of relatively greater performance in a prospective validation, in addition to the greater number of high quality data points upon which the model is trained upon.

**Table 1:** Dataset statistics.

**Strain**	**Synergistic combinations**	**Additive combinations**	**Antagonistic combinations**	**Total**
**TRAINING COMBINATIONS (HQ)**				
3d7	264 (22.1%)	762 (63.8%)	168 (14.1%)	1,194
Dd2	277 (22.2%)	817 (65.6%)	151 (12.1%)	1,245
Hb3	242 (20.9%)	767 (66.2%)	150 (12.9%)	1,159
**PROSPECTIVELY VALIDATED COMBINATIONS (HQ)**				
3d7	18 (15.1%)	100 (84%)	1 (0.8%)	119
Dd2	49 (26.5%)	134 (72.4%)	2 (1.1%)	185
Hb3	29 (35.8%)	52 (64.2%)	0	81

*Counts for the number of synergistic, additive, and antagonistic compounds in each of the datasets available for the current study, after filtering for high quality (HQ) data. The Dd2 training dataset had the highest number of HQ datapoints, which was reflected during cross validation (CV). The Dd2 dataset also contained the highest number of HQ datapoints in the prospectively validated dataset*.

The Dd2 dataset model was further examined in terms of the performance for each of the descriptor types, the results of which are displayed in Table 2. During 5-fold CV (where a random subset of 20% of the training data is held out to test upon), each descriptor type for Dd2 showed similar performance, with a cross-descriptor average MCC of 0.46 and a cross-descriptor average 2.78-fold enrichment (compared to random selection) of synergistic combinations correctly predicted by the model. However, for the more challenging leave-one-compound-out (LOCO) CV, the SFP model significantly outperformed the others, with MCC scores of 0.27 (SFP), 0.03 (TFP), and 0.03 (STFP). Moving on to the most difficult leave-one-pair-out (LOPO) CV routine, the performance was still greatest for the SFP model with a precision of 0.33 and recall of 0.01 (corresponding to an MCC of 0.02). Although recall (number of synergistic compounds in the test data that were identified correctly) is very low, the precision (number of synergistic combinations correctly identified in all that were predicted to be synergistic) is greater at 0.33. This is still useful in practice since it suggests *we are only likely to find the minority of all synergistic combinations in a dataset, but 33% of those combinations predicted to be synergistic will indeed turn out to be synergistic combinations*. Compared to our previous study where CoSynE was applied to antibiotic combinations (Mason et al., 2017), the LOPO CV performance was qualitatively similar with a high precision and low recall (1.0 and 0.2, respectively) for a SFP fingerprint on the training data. Since the coverage of chemical space in this dataset overall is quite low it is likely that the model has not been exposed to enough diversity to make confident predictions about many of the compounds, and so the recall score is low as a result.

**Table 2:** Dd2 training performance.

**CV**	**Descriptor**	**MCC**	**F1**	**AUC**	**Pr**	**Re**	**Ac**	**Ef**	**Rank**
5-Fold	SFP	0.45	0.56	0.84	0.61	0.53	0.82	2.74	2
	TFP	0.44	0.55	0.83	0.60	0.51	0.81	2.69	3
	STFP	0.47	0.57	0.84	0.64	0.52	0.83	2.89	1
	**Cross-descriptor average**	**0.46**	**0.56**	**0.84**	**0.62**	**0.52**	**0.82**	**2.78**	
LOCO	SFP	0.27	0.31	0.81	0.52	0.33	0.77	2.36	1
	TFP	0.03	0.08	0.58	0.07	0.11	0.76	0.31	3
	STFP	0.03	0.32	0.55	0.23	0.89	0.31	1.04	2
	**Cross-descriptor average**	**0.11**	**0.23**	**0.64**	**0.28**	**0.44**	**0.61**	**1.24**	
LOPO	SFP	0.02	0.01	0.44	0.33	0.01	0.78	1.50	1
	TFP	−0.02	0.10	0.49	0.20	0.07	0.73	0.89	3
	STFP	0.02	0.36	0.47	0.23	0.82	0.34	1.02	2
	**Cross-descriptor average**	**0.01**	**0.16**	**0.47**	**0.25**	**0.30**	**0.62**	**1.14**	

*The results from three increasingly difficult rounds of cross validation (CV); shuffled and stratified 5-fold CV, leave one compound out (LOCO), and leave one pair out (LOPO), for each model type (SFP, structural fingerprint; TFP, target fingerprint; and STFP, combined structure-target fingerprint). Since the current study concerns the prediction of novel compound combinations, our chosen model followed the expected performance of the SFP model during LOPO CV, since this is the most challenging test of the model. AUC, area under receiver operating curve; Pr, precision; Re, recall; Ac, accuracy; Ef, enrichment factor. The “cross descriptor average” is the average score for each metric across each cross validation routine*.

A possible reason behind the low performance of the TFP descriptor models is that the protein targets from PIDGIN are of human origin, and are unlikely to provide a useful representation of target interactions in *P. falciparum*. However, it is the case that orthologous proteins exist between *Homo sapiens* and *P. falciparum*, and it has previously been shown that the number of conflicting bioactivities between human and ortholog targets in public databases is comparatively low (Mervin et al., 2018), which supports the use of human targets as bioactivity spectra in this indirect manner. It has also been shown that bioactivity spectra can be used more generally as a descriptor that captures biologically relevant information, and can outperform chemical descriptors in the identification of compounds with similar bioactivities [see Petrone et al. (Petrone et al., 2012) Bender et al. (Bender et al., 2006), Kauvar et al. (Kauvar et al., 1995), Riniker et al. (Riniker et al., 2014), and Paricharak et al. (Paricharak et al., 2016)]. These, together with the lack of predictive modeling tools available to predict potential *P. falciparum* targets from a given compound structure, provided the reasoning behind our choice of entire bioactivity spectra against proteins as a descriptor type.

Since we are carrying out the toughest validation possible for our model by exploring novel areas of chemical space (i.e., the compounds to be prospectively validated in this study are not present in the training data), the most-challenging LOPO scenario represents the predictions we wish to make. The CV performance results suggest that by using the SFP descriptor model, we may expect an approximate 1.5-fold enrichment of synergistic combinations in those predicted from our novel compounds compared to random selection (although this enrichment appears low, note that there is already a high baseline of synergy within the dataset which this suggests could be increased further and that the prediction of synergy for entirely unseen data is the most difficult test of a predictive model possible). The SFP descriptor model was therefore selected as the most suitable candidate for this study, which is the same class of descriptor used in our previous study which successfully identified antibacterial combinations (Mason et al., 2017).

### Prospective validation of CoSynE predictions

The library of 23 compounds that were selected for prospective validation resulted from predictions generated by a developmental version of CoSynE that had previously virtually screened 21 million DrugBank combinations using the same training data, alongside a different approach that was developed in parallel to CoSynE (KalantarMotamedi et al., 2018; see Experimental section for details). From this library of 23 compounds (and a possible 253 combinations), a total of 20 combinations comprising 12 distinct individual compounds were predicted to be synergistic, and these were submitted for prospective experimental validation. The prospective validation found that 9 of these 20 combinations (i.e., 45%) exhibited antimalarial synergy (defined in this study as γ ≤ 0.96). These predicted synergistic combinations are shown in Table 3 where the range of γ is 0.917–0.958 (compared to the full prospective screen shown in Supplementary Table 2, where the range of γ is 0.88–0.959). The nine synergistic combinations that were correctly predicted comprise only seven compounds of the 23 that were provided to CoSynE. These seven compounds were further investigated using the literature, in order to identify a biological rationale for their selection, and are depicted in Table 4. It should be noted that five out of these seven compounds were found to also have self-self ɤvalues that would be classed as synergistic by the threshold that was trained upon, instead of additive (as one would expect). Inclusion of this observation in a predictive model would additionally include the experimental data for self-self crosses for all compounds, which may not be feasible. Instead, this highlights a current limitation of synergy quantification based upon experimental dose-response matrices, whereby the underlying metric should include these crosses as an additional parameter (see Experimental for details). In the present study however, the model has successfully predicted combinations of drugs that produced ɤvalues below a cutoff at a rate of 45%, demonstrating the ability to reduce search space significantly.

**Table 3:** Dd2 SFP predictions.

**Combination ID**	**Drug1 name (PubChem ID)**	**Drug2 name (PubChem ID)**	**Predicted probability of being synergistic**	**Prospectively derived γ (synergy ≤ 0.96)**
NCGC00167488 NCGC00021152	Sorafenib (216239)	Hydroxyzine (3658)	0.4	0.917
NCGC00263624 NCGC00017400	Apicidin (6918328)	Dihydroergotamine (10531)	0.42	0.924
NCGC00016272 NCGC00013226	Guanethidine (3518)	Trifluoperazine (5566)	0.36	0.926
NCGC00021152 NCGC00017400	Hydroxyzine (3658)	Dihydroergotamine (10531)	0.4	0.932
NCGC00167488 NCGC00013226	Sorafenib (216239)	Trifluoperazine (5566)	0.43	0.937
NCGC00181117 NCGC00017400	Virginiamycin s1 (46937022)	Dihydroergotamine (10531)	0.49	0.941
NCGC00263624 NCGC00021152	Apicidin (6918328)	Hydroxyzine (3658)	0.47	0.952
NCGC00263624 NCGC00181117	Apicidin (6918328)	Virginiamycin s1 (46937022)	0.62	0.957
NCGC00017400 NCGC00013226	Dihydroergotamine (10531)	Trifluoperazine (5566)	0.43	0.958

*The 9 combinations out of 20 predicted by CoSynE, which were prospectively validated to be synergistic, which cover a total of 7 unique compounds. The probability of being synergistic that was assigned by CoSynE is shown, which does not correlate with the experimentally quantified degree of synergy*.

**Table 4:** Synergistic drugs correctly predicted by CoSynE.

**Drug name**	**Depiction**	**Notes**
Apicidin	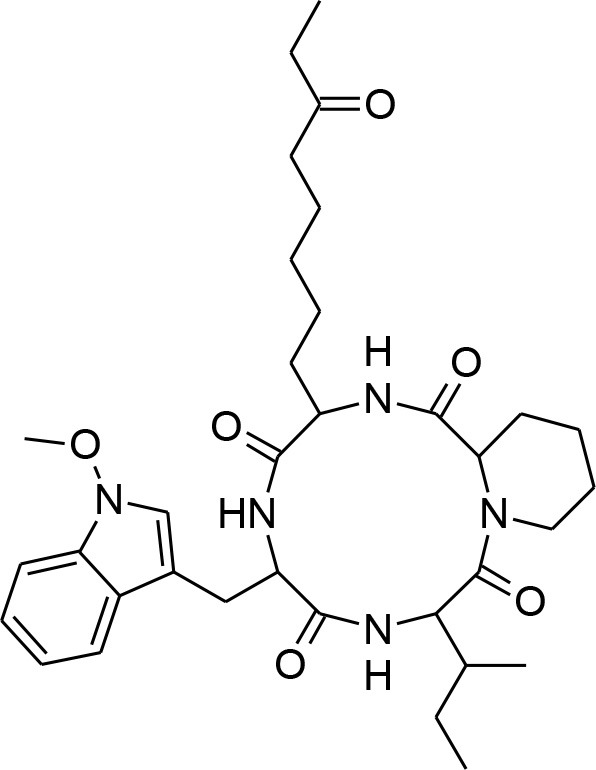	Known to target histone deacetylase and has previously shown activity against P. falciparum via inhibition of apicomplexan histone deacetylase (HDA) (Darkin-Rattray et al., 1996).
Dihydroergotamine	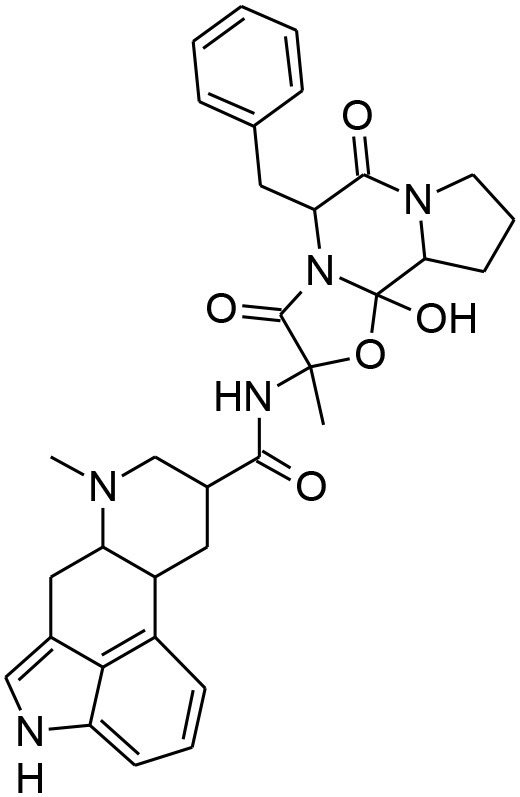	An inhibitor of P. falciparum (Weisman et al., 2006), and is annotated in PubChem as being active in several assays. May target a serotonin 5-HT1a-like receptor in the parasite thought to be a nutrient channel (Hanoun et al., 2003; Locher et al., 2003). Structural analog ergotamine achieved reasonably good docking score in a study searching for competitive inhibitors for *Pf*LDH (Penna-Coutinho et al., 2011).
Guanethidine	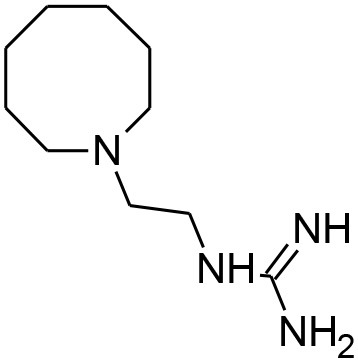	Annotated in PubChem as having an inconclusive potency against P. falciparum of 5.72 uM (AID:504834). Also annotated as active against MDR-1 (AID:377); the P. falciparum analog of which (pfmdr1) is involved in resistance and guanethidine may therefore play a role in preventing drug efflux (Hyde, 2007)
Hydroxyzine	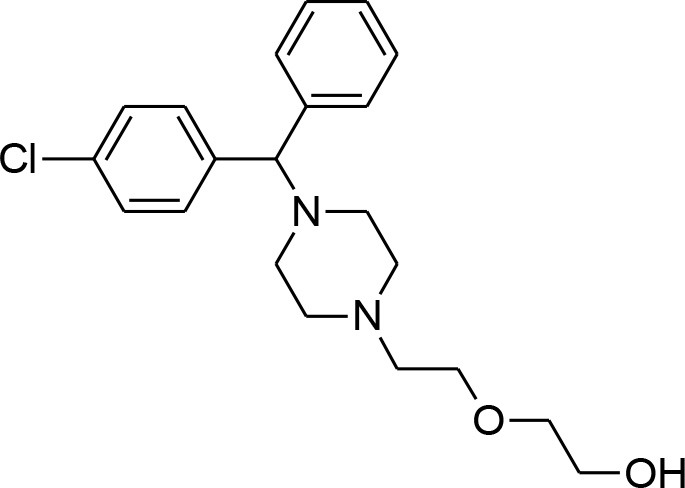	Shown to act as an efflux pump inhibitor in bacteria (Aybey et al., 2014). Also affects Quorum Sensing in microorganisms (Aybey et al., 2014).
Sorafenib tosylate	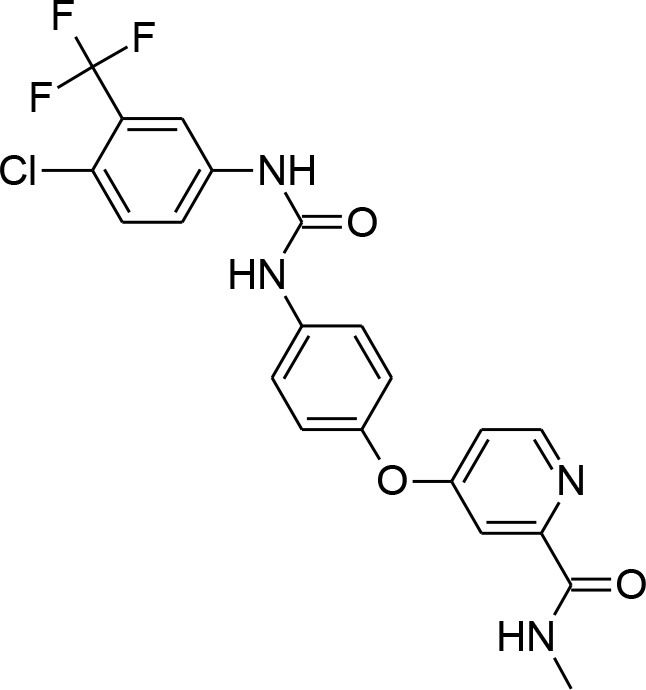	Tyrosine kinase inhibitor that exhibits antimalarial properties, and has been shown to inhibit the function of calcium-dependent protein kinase 3 in P. falciparum (PfCDPK1), which affects parasite egress from the host cell (Gaji et al., 2014). Sorafenib is an antitumor drug annotated in PubChem with activity against both 3D7 and DD2 strains, as well as RKL9, MRC2, and 7G8 with IC50s of 1.66–2.64 uM (Pathak et al., 2015). This compound was also tested in combination with artesunate in the study, however the mode of action was found to be antagonistic, while for another tyrosine kinase inhibitor, imatinib, combination with artesunate demonstrated synergy.
Trifluoperazine	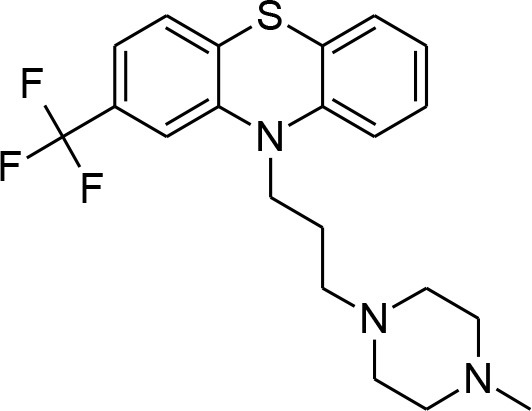	Calmodulin inhibitor, and a potent antiplasmodial inhibitor of calcium-dependent protein kinase 4 (PfCDPK4) (Cavagnino et al., 2011).
Virginiamycin s1	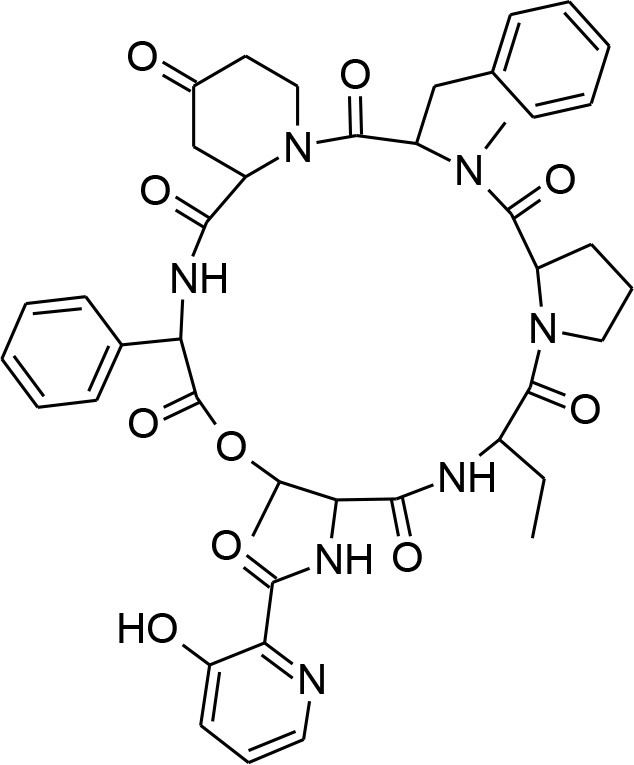	An antibiotic that is annotated as targeting 60S Ribosomal Protein L37 in PubChem. Similar in structure to azithromycin (which is known to target apicoplast 50S ribosomal subunit and inhibit P. falciparum).

*Depiction and description of the seven compounds that were part of combinations predicted to be synergistic by CoSynE*.

The following seven compounds were part of the nine combinations that were prospectively validated as being synergistic; dihydroergotamine (in four of the combinations), apicidin (three combinations), hydroxyzine (three combinations), trifluoperazine (three combinations), sorafenib (two combinations), virginiamycin factor S1 (two combinations), and guanethidine (one combination). The Tanimoto similarity of each compound vs. the training compounds is shown in Supplementary Figure 2, which shows apicidin has the greatest similarity among validation compounds to the training compounds at 39.1% (to gramicidin). Virginiamycin factor S1 is the next-closest compound to the training data, with a 30.7% similarity to gramicidin, followed by hydroxyzine (26.2% to piperaquine), trifluoperazine (24.6% to piperaquine), dihydroergotamine (23.5% to gramicidin), sorafenib (19.8% to nilotinib), and guanethidine (15.9% to pyronaridine). Overall, these greatest similarities to the training compounds are on the more-similar end of the distribution curve, but the overall similarity is still quite low. Compounds that form both the validation and training compounds are listed in Supplementary Tables 5, 7.

Out of the nine true positive synergistic predictions, four combinations involved one compound (namely, either hydroxyzine or guanethidine) known as a drug efflux pump inhibitor in other species (further details given below), which may also facilitate accumulation of a respective antimalarial partner drug in *P. falciparum*. Drug efflux pump inhibition has previously been suggested as attractive in combating resistance, whereby the intracellular concentration of an active compound is otherwise strongly restricted by the microorganism (Alibert-Franco et al., 2009). Firstly, hydroxyzine is a compound with antihistamine and central nervous system (CNS) properties that has been shown to act as an efflux pump inhibitor in bacteria, and also affects Quorum Sensing (QS) (Aybey et al., 2014). QS is a system of stimulus and coordination among microorganisms, which *P. falciparum* may use to detect conditions of the external environment (Wu et al., 2016), such as overcrowding, in order to keep the parasite population under control in the host (Mutai and Waitumbi, 2010). Hydroxyzine was correctly predicted to be synergistic in combination with sorafenib, apicidin, or dihydroergotamine. Sorafenib is a tyrosine kinase inhibitor used in the treatment of cancer that inhibits parasite egress from the host cell (Gaji et al., 2014), and is annotated with activity against both 3D7 and Dd2 strains of *P. falciparum* in PubChem (Pathak et al., 2015; Kim et al., 2016). Apicidin is a potent inhibitor of histone deacetylase [HDA; of which the *P. falciparum* ortholog PfHDA2 exists (Coleman et al., 2014)] and this mechanism of inhibition is responsible for the antiprotozoal properties of the drug (Darkin-Rattray et al., 1996; Engel et al., 2015). Dihydroergotamine is a known inhibitor of *P. falciparum* (Weisman et al., 2006), which may target a serotonin 5-HT1a-like receptor in the parasite thought to be a nutrient channel critical for parasite development (Hanoun et al., 2003; Locher et al., 2003). Ergotamine, the structural analog of dihydroergotamine was one compound involved in a docking study looking for competitive inhibitors for the enzyme *P. falciparum* lactate dehydrogenase (*Pf* LDH), upon which the parasite is dependent for energy production where it achieved a reasonably good docking score (Penna-Coutinho et al., 2011). The combination of these active compounds with the hydroxyzine efflux pump inhibition and QS action may be responsible for the observed synergy in these cases. Secondly, guanethidine is annotated as active against human multidrug resistance protein 1 (MDR-1) in a screen for compounds that compete for this transporter as a means to increase accumulation of active compounds in cells (AID:377). A plasmodium ortholog of MDR-1, PfMDR1 exists (Hyde, 2007), and if guanethidine competes for PfMDR1, this may explain a potential mechanism for synergy, since PfMDR1 is important for transporting substrates from the cytoplasm into the lysosomal-like parasite digestive vacuole (Reiling and Rohrbach, 2015). Guanethidine alone does not show activity against *P. falciparum* (Chong et al., 2006), but was correctly predicted to show synergy in combination with trifluoperazine. Trifluoperazine is an antipsychotic drug and a potent inhibitor of *P. falciparum* calcium-dependent protein kinase 4 (PfCDPK4) (Cavagnino et al., 2011), and so would represent the anti-malarial compound in this combination. To the authors' knowledge, these may be novel modes of action for the use of hydroxyzine and guanethidine in context of *P. falciparum*. Since the training dataset did not include compounds explicitly annotated as targeting *P. falciparum* efflux pumps [with the exception of primaquine, which exhibits synergy with chloroquine through inhibiting the *P. falciparum* Chloroquine Resistance Transporter; PfCRT (Bray et al., 2005)]. Further experimental validation would be required to confirm this mechanistic hypothesis of the synergies observed experimentally.

Three of the remaining five combinations that were correctly predicted involve a combination of the previously detailed compounds that were the “active” partner drugs to those with expected efflux pump inhibitors (apicidin-dihydroergotamine, trifluoperazine-sorafenib, and trifluoperazine-dihydroergotamine). The observed synergy in these may exert their synergistic effect through their differing mechanisms.

The final two correctly predicted combinations involve virginiamycin factor S1, a macrolide antibiotic annotated as active against *P. falciparum* proliferation (AID:504749), with either apicidin or dihydroergotamine. Antibiotics may exhibit antimalarial properties, albeit slow-acting, by targeting the apicoplast during development (Dahl et al., 2006; Barthel et al., 2008; Chakraborty, 2016). Macrolides are known for their effectiveness in treatment of uncomplicated malaria in combination with quinine, where the main mechanism of action involves binding to ribosomal proteins, but suffer due to poor pharmacological properties (Gaillard et al., 2016). The combination of virginiamycin S1 targeting the apicoplast, and apicidin targeting plasmodium orthologs of histone deacetylase, such as PfHDA2 (Darkin-Rattray et al., 1996; Coleman et al., 2014; Engel et al., 2015) suggests that this combination puts pressure on the developmental and growth stages of the parasite. The combination of potential nutrient channel and energy inhibition properties of dihydroergotamine (Hanoun et al., 2003; Locher et al., 2003; Penna-Coutinho et al., 2011) with the apicoplast-targeting mechanism of virginiamycin S1 also suggests pressure being put on the developmental and growth stages. However, since this work used asynchrous parasite cultures to assess compound efficacy, and given that apicoplast-targeting molecules don't typically affect the first replication cycle upon drug pressure [where they are instead exhibiting a “delayed death” phenotype (Dahl and Rosenthal, 2007)], this apicoplast-targeting mechanism is unlikely to have been observed. Unfortunately, the combination of macrolides and dihydroergotamine has been reported to produce clinically significant adverse drug reactions (Horowitz et al., 1996), which means this particular combination would not be suitable as a potential treatment.

### Full pairwise synergy screen of 23 compounds

A subsequent full pairwise experimental screen of all 23 compounds was also carried out (Supplementary Table 3), in order to assess the performance of CoSynE for the prediction of completely novel combinations of compounds acting synergistically. Comparison of the overall number of synergistic combinations that were found (49 out of 185, or 26%, see Table 1), compared to the number that was present among those predicted by CoSynE (9 out of 20, or 45%) showed that we achieved a 1.70-fold enrichment (0.45/0.265); approximately that which was expected from our LOPO CV performance. This level of enrichment is significant in the search for antimalarial compound combinations in practical terms, where a 41% reduction [1 – (1/1.70)] in the total number of measurements required is a very attractive prospect in terms of both time and cost. Although this performance is attractive, the model is still far from ideal and requires further refinement to increase both the precision (0.45) and recall (0.18) seen in Table 5. On the other hand it should be noted that the baseline of obtaining synergy in 26.5% of cases is a rather high baseline, which the model was able to increase further to nearly half of all synergistic predictions being true positives (more precisely, to 45% of all combinations).

**Table 5:** Dd2 SFP Performance.

**Descriptor**	**Predicted synergistic combinations**	**Experimentally validated as synergistic**	**MCC**	**F1**	**AUC**	**Pr**	**Re**	**Ac**	**Ef**
SFP	20	9	0.15	0.26	0.63	0.45	0.18	0.72	1.70

*Overall performance of the Dd2 SFP model, after the full pairwise screen of prospective compounds was carried out. Overall, the precision and recall for the prediction of novel synergistic combinations, however this still provides greater enrichment of synergistic combinations than expected by random selection (1.70-fold) from the prospectively validated dataset. AUC, area under receiver operating curve; Pr, precision; Re, recall; Ac, accuracy; Ef, enrichment factor*.

### Potential for indirect discovery of synergistic combinations

We next investigated the hypothetical scenario where all compounds that are part of combinations predicted to be synergistic by CoSynE were screened in a fully pairwise manner, to see whether CoSynE could indirectly expand the discovery of novel combinations. Interpreted differently, we investigated whether synergy between compounds is “clustered”—and whether the knowledge that a compound has shown synergy before increases the chances that it will show synergy also in combination with other compounds (with the limitation of our validation being the limited sampling of chemical space, which may or may not generalize to “all” chemical space). Each combination in the prospective validation dataset for Dd2 involving any of the 12 compounds that were part of a combination predicted to be synergistic was extracted, yielding a total of 61 combinations, out of which 36% were found to be synergistic (22 combinations in Supplementary Table 4). This proportion of synergistic combinations is hence higher (by 9.5% in absolute terms, and 36% in relative terms) than the 26.5% found in all of the 185 HQ validation combinations, which corresponds to an enrichment of 1.36 × compared to random selection. However, to some extent this enrichment may be slightly inflated due to CoSynE having identified drug efflux pump inhibitors in the model. Among the synergistic combinations in this subset indirectly found through CoSynE is guanethidine (antiplasmodial and active against MDR1) and paroxetine (annotated in DrugBank as targeting MDR1, antibacterial activity via efflux pump and QS inhibition Aybey et al., 2014, and antiplasmodial activity Chong et al., 2006 including AID:524790–524796), with a ɤscore of 0.889. This combination is more synergistic than all those directly predicted by CoSynE, and is the second-most synergistic combination among all HQ combinations in the validation dataset. This suggests that by not only screening compound combinations predicted to be synergistic by CoSynE, but *all combinations* of the compounds predicted to be part of *any* combination predicted to be synergistic will still increase the likelihood of identifying further synergistic combinations. This also is in line with previous studies, which have found that while synergy to an extent depends on the properties of both compounds in a combination, there is still a significant bias in chemical space, with some parts of it being significantly more frequently part of synergistic compounds combinations than others (Weinstein et al., 2017).

Along these lines, we believe that an iterative screening procedure could be followed in an industrial setting, whereby predictions are made, screened, and then fed back into CoSynE for training before further predictions are made. Such iterative approaches have been investigated in the literature (Paricharak et al., 2016), and could enable gradual expansion of chemical and/or biological space, in particular with current improvements in cherry picking compounds in such iterative screening settings.

## Conclusion

In this work, we describe the application of our compound combination prediction method, CoSynE, to a recently published compound combination screening dataset for *P. falciparum*, and the results to a prospective validation of our predictions. When we used our final CoSynE model to predict synergistic combinations (γ ≤ 0.96) from a library of compounds previously unknown to the model for *P. falciparum* Dd2, 45% of the predicted combinations (9 out of 20) were experimentally confirmed as being synergistic, corresponding to a 1.70-fold enrichment of synergistic combinations than that expected by randomly selection from the validation dataset. This is of practical significance when combinatorial explosion and experimental cost for combination screening is taken into account. Furthermore, a 2.36-fold enrichment was observed during cross validation when one compound is unknown, and 2.74-fold when both compounds are known to the model (but only in different combinations). In addition, it was found that screening only compounds part of combinations CoSynE predicted to be synergistic would yield 9.5% more synergistic combinations in absolute terms (and 36% in relative terms) than expected by random selection alone.

The combinations that were prospectively validated from our predictions mainly involve one compound with antimalarial activity coupled to another targeting potential drug efflux or substrate transport mechanisms in *P. falciparum*. These results in particular suggest that the approach we describe can capture meaningful information that enables the prediction of synergy, which is corroborated by our previous study involving antibiotic combinations.

CoSynE offers an advantage over similar methods that require data, such as differential gene expression analysis, or single agent efficacies across multiple cell lines related to the target, in that the only information required to make new predictions is the provision of chemical structure information. The use of CoSynE to make predictions for other therapeutic areas requires only a dataset of combination screening results together with compound structural information, and may also predict for higher orders of combinations (e.g., combinations of 3, 4, and above), should training data with a meaningful measure of synergy be made available. Our approach may be employed to prioritize screening of new combinations, thus reducing the potential burden and cost of combinatorial explosion in the search for future antimalarial compound combinations that exhibit synergy.

## Experimental

### Experimental screening of compound combinations

Training data was obtained from a publicly available dataset of antimalarial compound combinations from a high-throughput screen against 3D7, Dd2, and HB3 strains of *P. falciparum* (assay IDs 1463, 1464 and 1465, which can be found at https://tripod.nih.gov/matrix-client/?p=183; Mott et al., 2015). Compounds were acoustically dispensed and read at 72 h as previously described (Mott et al., 2015). Matrix combination response was calculated based upon relative SYBRGreen intensity values, compared to controls (Mott et al., 2015). The prospective validation data was screened using the same method as the training data. This validation dataset includes both single-agent and combination responses, and can be found at https://tripod.nih.gov/matrix-client/?p=1261. The 23 compounds that comprised the validation dataset are listed in Supplementary Table 5, the experimental data used to validate the Dd2 model is listed in Supplementary Table 3, and reproducibility of assay results is detailed in Supplementary Table 6.

### Compound combination datasets and synergy

The training data used in this study consisted of 1,540 combinations of 56 antimalarial compounds that exhibit different modes of action, which were screened against the 3D7, Dd2, and HB3 strains of *P. falciparum*. The 56 compounds that formed this screen are listed in Supplementary Table 7. Synergy metrics and data quality (QC) were pre-determined from a 6 × 6 dose-response matrix of each combination, where inhibition of the parasite in infected red blood cells was measured. The QC score for a combination was precomputed from a set of heuristics described in Mott et al. (2015), that takes in to account the quality of the single agent dose response, DMSO activity and the smoothness of the dose combination response matrix. This yields a value between 0 and 18, where lower values indicate higher quality. Only high quality (HQ) experimental readouts were kept that have a QC score ≤ 3, which provided 1,194 HQ combinations for 3D7, 1,245 for Dd2, and 1,159 for HB3 (Table 1; training dataset). For the validation dataset, the same filtering rules applied to 209 combinations of 23 compounds provided 119 for 3D7, 185 for Dd2, and 81 for HB3 (Table 1; validation dataset).

The metric used to interpret synergy in our modeling approach was gamma (ɤ), which is a combination of the Highest Single Agent (HSA; also known as Gaddum's non-interaction model) and Bliss independence. Based upon a 6 × 6 dose-response matrix of compound A and compound B at concentration *x* and *y* vs. inhibition of *P. falciparum*, the variable ɤis computed to minimize the following function (Cokol et al., 2014).

(1) $$\begin{array}{l} \Sigma {\left[f\left(A_{\left[x\right]}+\;B_{\left[y\right]}\right)-\;\gamma \;\times \;max\left\{f\left(A_{\left[x\right]}\right),\;f\left(\;B_{\left[y\right]}\right)\right\}\right]}^{2} \end{array}$$

This yields a positive value, where synergy is characterized as <1, additivity as = 1, and antagonism as >1. In order to classify each of the combination readouts, we set a maximum ɤcutoff for synergy of 0.96, and minimum cutoff for antagonism of 1.04, with the remainder assigned as additive. This cutoff value was empirically chosen to provide a degree of separation between antagonism and synergy in the training data, while aiming to keep the balance of each class similar across strains. Although not explicitly investigated during the study, we expect that making the ɤcutoff larger may lead to an increased enrichment of synergistic combinations being predicted, while making it smaller may affect the model robustness by decreasing the number of synergistic training datapoints further.

One limitation with regard to the pre-processing of experimental combination responses during our study is that measurement of self-crosses using the Bliss model component of ɤmay in fact produce values which are classed as synergistic. For example, apicidin in combination with itself in the validation dataset shows a ɤvalue of 0.895, whereas our cut-off for the training data was 0.96. In other words, this self-cross should be labeled as “synergistic” according to our criteria, whereas self-interaction should be additive; this is a well-known phenomenon among synergy measures, where a generalizable and robust model is yet to be identified (Bulusu et al., 2016). We chose to apply the cut-off of 0.96 that was used for the training data to enable our assessment of validation predictions “in the eyes of the model” with respect to training criteria, yielding 49 synergistic combinations in the Dd2 validation dataset. Compounds with self-cross ɤvalues lower than our training data cut-off include trifluoperazine, raloxifene, guanethidine, hydroxyzine, megestrol acetate, FK-506, fulvestrant, sorafenib, apicidin, and ingenol mebutate. Since these cover five out of the seven compounds in Table 3, any future investigation into combinations involving these compounds based solely upon ɤvalues should bear this in mind (i.e., eight out of our nine predictions in Table 3). Although it is not clear precisely how to overcome this limitation, future models that additionally train upon the validation dataset might take these self-crosses into account more explicitly by lowering synergistic cut-offs on a per-combination basis, or seek to find a way of incorporating this into the synergy metric itself. All self-crosses for the validation data may be found at https://tripod.nih.gov/matrix-client/?p=1261, and minimum significance ratios for the validation compounds that were screened are detailed in Supplementary Table 6.

### Prior selection of validation compounds

The selection of compound combinations for screening and validation of our models were based upon a version of CoSynE much earlier in development. Several CoSynE models were trained upon the same dataset as described in this report, except the range of additivity for ɤwas narrower at 0.975–1.025 (opposed to 0.96–1.04). The resulting models were used to predict enumerated combinations of approved, investigational, and experimental compounds in DrugBank (Wishart et al., 2006), which amounted to around 21 million combinations for prediction. Of these, approximately 1.2 million combinations were predicted to be synergistic, and 10 combinations needed to be selected for the prospective validation. This selection was achieved by manually reviewing the top-ranked combinations (sorted by the probability of being synergistic that was assigned to each combination by CoSynE), and taking into consideration the prevalence of each compound throughout the list of combination predictions, followed by examining the literature co-occurrence of each predicted combination's compounds together with mention of *P. falciparum* in PubMed. These 10 chosen combinations comprised 18 compounds, and were submitted for testing together with an additional 10 selected from a different approach developed in parallel by KalantarMotamedi et al. (2018).

Out of the total number of compounds among the 20 combinations primarily suggested for testing, only the 23 compounds shown in Supplementary Table 5 were available for purchase at the time, which meant few original predictions could be prospectively validated. The decision was made to instead use a more recent version of CoSynE to predict which combinations of these 23 compounds were synergistic, finally yielding the dataset in this study. Interestingly, Table 1 shows that the number of antagonistic combinations observed in the validation dataset is significantly lower compared to the training dataset, while at the same time the number classed as additive or synergistic has increased. This reduction in the number of antagonistic combinations as a result of virtually screening a library of intractable size suggests that the approach taken by CoSynE, together with the process of manually reviewing the top predictions, aids the discovery of synergistic combinations.

### Comparison to a similar study conducted in parallel

The approach by KalantarMotamedi et al. (2018) differs from that described in this work primarily by the usage of gene expression data. Firstly, differential gene expression profiles of mild vs. severe malaria patient peripheral blood samples were used to predict potentially active single antimalarial agents by comparison of drug gene perturbations through a modified Gene Set Enrichment Analysis (GSEA) approach (Subramanian et al., 2005) applied to the Library of INtegrated Cellular Signatures (LINCS) Phase I database (Subramanian et al., 2017). Secondly, a Random Forest model was trained on the same dataset of 1,540 combinations from NCATS as in the present study, and human target predictions and pathway annotations were used to infer which drug combinations may interact synergistically. Finally, the single agents identified by the GSEA approach to human blood samples were enumerated as pairs and predicted by the Random Forest model as synergistic/non-synergistic. These predicted combinations were ranked based upon the predicted probability of being synergistic, and the top 17 compound combinations were selected for prospective experimental testing (covering a total of 14 single agents). This approach reported an overall average precision of 0.488 and recall of 0.755 (F1 = 0.593) for experiments across the three strains of *P. falciparum* where drug combinations were predicted to be synergistic at a cutoff for synergy of γ ≤ 0.975. Among the 14 single agents in 17 combinations Kalantar-Motamedi et al. selected for prospective validation were seven that overlapped with the 12 drugs in 20 combinations CoSynE predicted for prospective validation; ciprofloxacin, wortmannin, paroxetine, raloxifene, apicidin, trifluoperazine, and hydroxyzine. The only combination of these overlapping compounds that was correctly predicted to be synergistic in both CoSynE and the method described by Kalantar-Motamedi et al. was apicidin-hydroxyzine. Since CoSynE is not constrained to compounds that are only present in the Connectivity-Map (Lamb et al., 2006) or LINCS databases (instead needing only knowledge of compound structure) it is difficult to draw a direct and fair comparison of overall performance. However, for the same experimental γ cutoff applied to the total pool of 185 prospective combinations in the current study that denotes a synergistic combination, CoSynE achieved precision of 0.45 and recall of 0.18 (F1 = 0.26). While the precision of CoSynE for the prospectively validated combinations is close to that reported by Kalantar-Motamedi et al. recall in this instance is much lower. However, it should be noted this overall performance still represents greater enrichment of synergistic combinations being discovered than by random selection (see Table 5), and CoSynE is not limited by the requirement for gene expression data to be made available for the compounds that are to be predicted.

### Combination descriptors

We represented each compound combination as an array of features in three ways. A Structural Fingerprint (SFP) descriptor based upon the molecular structure of each compound in a combination, a Target Fingerprint (TFP) descriptor based upon probabilistic combination of predicted target affinity probabilities per compound, and a concatenation of these two previous descriptors (Structure-Target; STFP). This provided three descriptor sets for which models were trained.

Structural fingerprints were generated by first obtaining SMILES representation PubChem (Kim et al., 2016) for each compound that was screened in the training data, before standardizing this representation with ChemAxon JChem Standardizer (ChemAxon, 2014) according to the protocol defined by PIDGIN (Mervin et al., 2015). Standardized SMILES were then loaded into RDKit v2015^1^ and 2,048-bit Morgan fingerprints with radius 2 were generated, yielding arrays of 2,048 integer features. A given combination of two compounds was represented as the bitwise average of these features, yielding possible values of 0, 0.5, and 1 per feature, which formed the SFP descriptor. A Morgan fingerprint was chosen for this study due to generally outperforming the MACCS fingerprint in this dataset [however the MACCS fingerprint was found to outperform Morgan when CoSynE was used to predict antibiotic combinations (Mason et al., 2017)]. The SMILES representation was also used as input for PIDGIN (Mervin et al., 2015), where the probability of binding below the training cut-off of 10 μM for each compound vs. 1,080 human protein targets was predicted, yielding arrays of 1,080 floating point value features between 0 and 1. A given combination considered the probability of binding to each protein target by each compound from the following function, such that the maximum affinity a combination of compounds may have is 100% [i.e., a value of 1.0; Equation (2)], which formed the TFP descriptor. The rationale behind the use of this function for TFP was that the probability of a protein being inhibited cannot be more than 100%, but the more compounds in a single combination that are predicted to target the protein, the more this is likely to be the case.

(2) $$\begin{array}{l} p\left(Combination,\;TargetN\right)=1-\left(1-p\left(Compound1,\;TargetN\right)\right)\\ \times \left(1-p\left(Compound2,\;TargetN\right)\right) \end{array}$$

### Model construction and performance testing

Model settings were optimized prior to construction of the final models, and all machine learning capabilities were carried out using SciKit-Learn v0.17 (Pedregosa et al., 2011).

The 1,245 Dd2 compound combinations that formed our training data each has either between 1,080, 2,048, or 3,128 features per combination (depending on the descriptor used), meaning that the feature space is larger than the number of combinations. It is therefore necessary to remove any features that are not useful for training prior to constructing the final models. Training data was scaled to unit variance with a zero-centered mean, and starting from *N* = 1, the top *N* percentile of features within the training data [as determined by ANOVA F-classifier score in SciKit-Learn v0.17 (Pedregosa et al., 2011)] was selected to train upon using a Support Vector Machine Classifier (SVC, optimization parameters detailed in Supplementary Methods), together with the synergy type labels per combination, to construct a classifier. This classifier then predicted the synergy label for test data that has had the same features selected, and the outcome of this test was scored using the Matthews Correlation Coefficient [MCC, Equation (3)] with respect to the ability for correctly predicting a synergistic combination. Due to the consideration of all possible outcomes of a classification problem (true positive; TP, false positive; FP, true negative; TN, false negative; FN), the MCC score offers benefit over performance metrics, such as the Area Under Receiver Operating Curve (AU-ROC) and Accuracy, which ignore TN and TN, and FP and FN predictions, respectively.

(3) $$\begin{array}{l} MCC=\;\frac{TP\;\times TN-FP\;\times FN}{\sqrt{\left(TP+FP\right)\left(TP+FN\right)\left(TN+FP\right)\left(TN+FN\right)}} \end{array}$$

This process was repeated 10 times per N, by stratified and shuffled 5-fold cross validation, to finally yield 99 averaged MCC scores. These top *N* selected features that resulted in the highest MCC score overall were subsequently used by CoSynE in the final model training round, in order to test model performance in different scenarios. The top *N* selected features per model are detailed in Supplementary Methods. While CoSynE will label predicted combinations as synergistic, additive, or antagonistic, during model optimization only the prediction of synergistic combinations is carried out.

The second round that results in selection of the final model involved construction of a number of different classifiers [Bernoulli Naïve Bayes, Support Vector Machine, Random Forest, Extra Trees, and Decision Tree, SciKit-Learn v0.17 (Pedregosa et al., 2011)], which were subject to grid search parameter optimization (optimization parameters detailed in Supplementary Methods). The selection of the best model parameters was based upon 10 repeats of stratified and shuffled 5-fold cross validation, which represents a scenario where the training data has prior knowledge of both compounds per combination (Figure 3). Each model with a new set of parameters was then subjected to two further rounds of validation of increasing difficulty; Leave One Compound Out (LOCO; in which one compound in a combination is made unknown to the model), and Leave One Pair Out (LOPO; in which both compounds are made unknown to the model). This provided a view on model performance when looking to extend the compounds used in combination with those already known (LOCO) or, in the toughest case, searching for novel combinations of unknown compounds (LOPO). The choice of final model settings was based upon performance in terms of the MCC score for the prediction of synergistic combinations in each of these scenarios.

In each test and train split of the data, feature selection and scaling were based solely upon the training data to ensure that no information from the test set was used in the model generation step. Final model settings are detailed in Supplementary Table 7.

## Author contributions

DM created the tool and wrote the majority of the manuscript. RL provided advice with respect to the training dataset. RG and RE carried out the experimental work. IS and AB obtained funding, supervised and provided advice.

### Conflict of interest statement

DM was employed by company Healx Ltd. at the time of submission. IS was employed by company Unilever at the time of submission. RG was employed by company Vertex Pharmaceuticals at the time of submission. The remaining authors declare that the research was conducted in the absence of any commercial or financial relationships that could be construed as a potential conflict of interest.

## Footnotes

^1^Landrum, G. *RDKit: Open-Source Cheminformatics*. Available online at: http://www.rdkit.org
