# Supplementary material for: Using Machine Learning to Predict Synergistic Antimalarial Compound Combinations With Novel Structures
Source: Front Pharmacol. 2018 Oct 2;9:1096. doi: 10.3389/fphar.2018.01096 (PMC6176478; doi:10.3389/fphar.2018.01096)
Supplement: Supplementary file 2 [file Data_Sheet_2.DOCX]

**SI - Hyperparameters and final model settings used to train CoSynE. These parameters in JSON format can be re-loaded back into SciKit-Learn**

**# Hyperparameters used for feature selection:**

{'svm__kernel':['sigmoid', 'rbf'], 'svm__gamma':[1e-6, 1e-5, 0.0001, 0.001, 0.01, 0.1, 1.0],'svm__C':[0.1, 1., 10., 100., 1000., 10000.]}

**# Hyperparameters used to determine best predictive algorithm:**

classifier_hyperparams = {

'svm': {'model__kernel':['rbf'],'model__gamma':[1e-6, 1e-5, 0.0001, 0.001, 0.01, 0.1, 1.0], 'model__C':[0.1, 1., 10., 100., 1000., 10000.]},

'rf': {'model__n_estimators':[500, 1000, 2000],"model__criterion": ["gini", "entropy"],'model__max_features':[None, 'auto', 'sqrt', 'log2'],'model__min_samples_split':[1, 2, 3]},

'et': {'model__n_estimators':[500, 1000, 2000],"model__criterion": ["gini", "entropy"],'model__max_features':[None, 'auto', 'sqrt', 'log2'],'model__min_samples_split':[1, 2, 3]},

'bnb': {'model__alpha':[1.,2.,3.],'model__binarize':[0., 1.,2.,3.]},

'dt': {'model__min_samples_leaf':[1, 2, 3],'model__max_depth':[None, 1, 2, 3],'model__max_features':[None, 'auto', 'sqrt', 'log2']},

'knn': {'model__n_neighbors':[5,10,20], 'model__weights':['uniform', 'distance'], 'model__algorithm':['auto', 'ball_tree', 'kd_tree', 'brute'], 'model__leaf_size':[15,30,45], 'model__p':[1,2,3]}

}

**# Parameters used in the final CoSynE models, used to make predictions in the article:**

**Descriptor type: Combined**

**Settings for model: SVC**

{'kernel': 'rbf', 'C': 100.0, 'verbose': False, 'probability': True, 'degree': 3, 'shrinking': True, 'max_iter': -1, 'decision_function_shape': None, 'random_state': None, 'tol': 0.001, 'cache_size': 200, 'coef0': 0.0, 'gamma': 0.0001, 'class_weight': None}

**Settings for scaler:**

{'copy': True, 'with_mean': True, 'with_std': True}

**Settings for anova:**

'percentile:' 68.00, 'score_func:' f_classif

**Descriptor type: PredictedTargets**

**Settings for model: SVC**

{'kernel': 'rbf', 'C': 10000.0, 'verbose': False, 'probability': True, 'degree': 3, 'shrinking': True, 'max_iter': -1, 'decision_function_shape': None, 'random_state': None, 'tol': 0.001, 'cache_size': 200, 'coef0': 0.0, 'gamma': 1e-05, 'class_weight': None}

**Settings for scaler:**

{'copy': True, 'with_mean': True, 'with_std': True}

**Settings for anova:**

'percentile:' 97.00, 'score_func:' f_classif

**Descriptor type: StructuralFingerprint**

**Settings for model: SVC**

{'kernel': 'rbf', 'C': 10000.0, 'verbose': False, 'probability': True, 'degree': 3, 'shrinking': True, 'max_iter': -1, 'decision_function_shape': None, 'random_state': None, 'tol': 0.001, 'cache_size': 200, 'coef0': 0.0, 'gamma': 1e-05, 'class_weight': None}

**Settings for scaler:**

{'copy': True, 'with_mean': True, 'with_std': True}

**Settings for anova:**

'percentile:' 49.00, 'score_func:' f_classif
