# Supplementary material for: Using Machine Learning to Predict Synergistic Antimalarial Compound Combinations With Novel Structures
Source: Front Pharmacol. 2018 Oct 2;9:1096. doi: 10.3389/fphar.2018.01096 (PMC6176478; doi:10.3389/fphar.2018.01096)
Supplement: Supplementary file 3 [file Image_1.PDF]

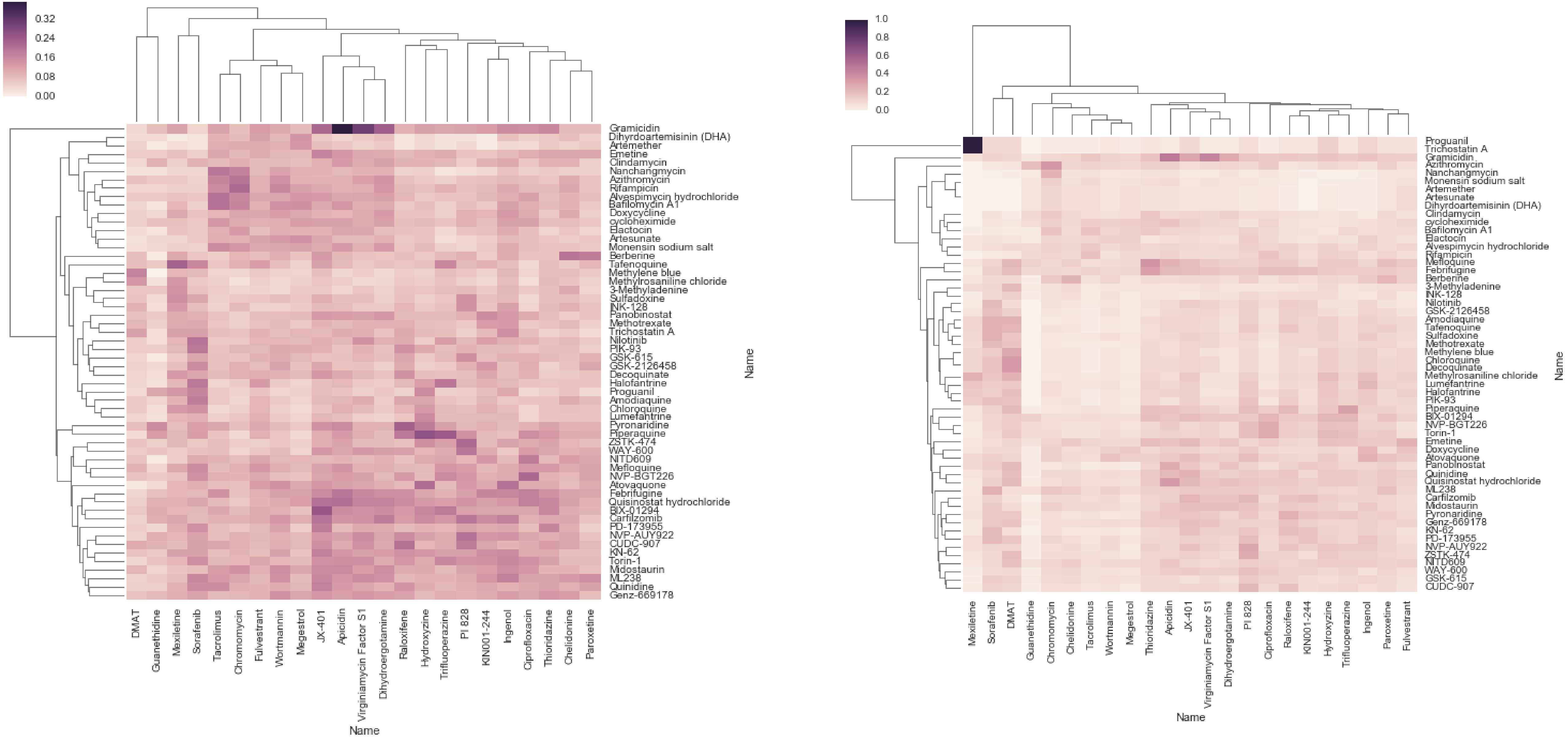

SI Figure 1. Hierarchically clustered molecular similarity matrices for compounds that formed the training data and prospectively validated test data. Left; similarity based upon whole chemical structures. Right; similarity based upon Murcko scaffold structures. Similarity was calculated by the Tanimoto coefficient for 2048-bit r=2 Morgan fingerprints. In general there is little similarity between compounds in the train and test datasets.

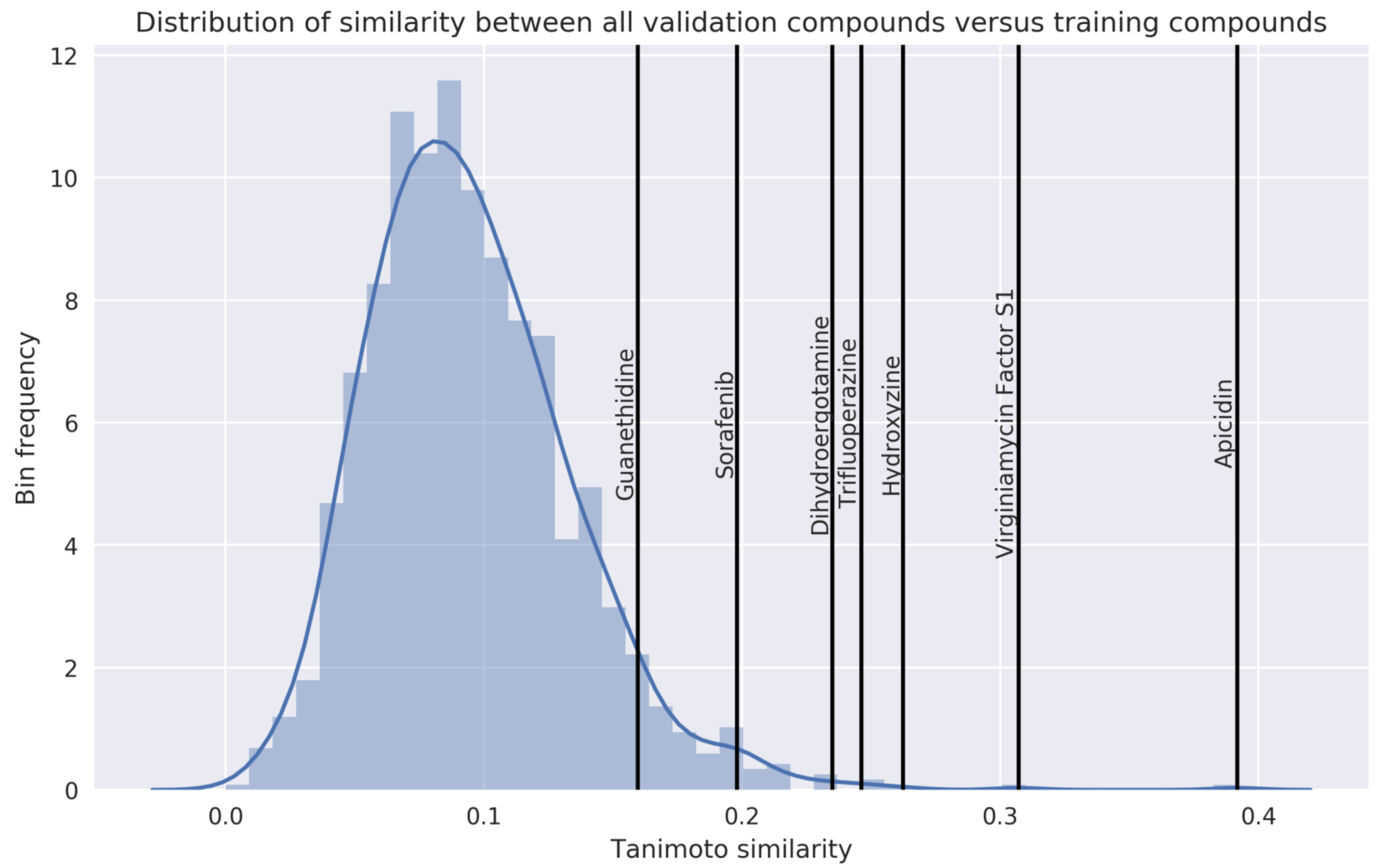

SI Figure 2. A distribution of validation compound similarities versus training compounds similarities. Highlighted vertically are the 7 compounds that were part of correctly predicted synergistic combinations after prospective validation. Similarity was calculated by the Tanimoto coefficient for 2048-bit r=2 Morgan fingerprints. In general these compounds are quite similar to some of the training compounds, but this similarity is quite low.
